# Microbiome of the Successful Freshwater Invader, the Signal Crayfish, and Its Changes along the Invasion Range

**DOI:** 10.1128/Spectrum.00389-21

**Published:** 2021-09-08

**Authors:** Paula Dragičević, Ana Bielen, Ines Petrić, Marija Vuk, Jurica Žučko, Sandra Hudina

**Affiliations:** a Department of Biology, Faculty of Science, University of Zagreb, Zagreb, Croatia; b Faculty of Food Technology and Biotechnology, University of Zagreb, Zagreb, Croatia; c Ruer Bošković Institute, Zagreb, Croatia; Howard University

**Keywords:** invasive species, *Pacifastacus leniusculus*, 16S rRNA gene, microbiome, range expansion

## Abstract

Increasing evidence denotes the role of the microbiome in biological invasions, since it is known that microbes can affect the fitness of the host. Here, we demonstrate differences in the composition of an invader’s microbiome along the invasion range, suggesting that its microbial communities may affect and be affected by range expansion. Using a 16S rRNA gene amplicon sequencing approach, we (i) analyzed the microbiomes of different tissues (exoskeleton, hemolymph, hepatopancreas, and intestine) of a successful freshwater invader, the signal crayfish, (ii) compared them to the surrounding water and sediment, and (iii) explored their changes along the invasion range. Exoskeletal, hepatopancreatic, and intestinal microbiomes varied between invasion core and invasion front populations. This indicates that they may be partly determined by population density, which was higher in the invasion core than in the invasion front. The highly diverse microbiome of exoskeletal biofilm was partly shaped by the environment (due to the similarity with the sediment microbiome) and partly by intrinsic crayfish parameters (due to the high proportion of exoskeleton-unique amplicon sequence variants [ASVs]), including the differences in invasion core and front population structure. Hemolymph had the most distinct microbiome compared to other tissues and differed between upstream (rural) and downstream (urban) river sections, indicating that its microbiome is potentially more driven by the effects of the abiotic environment. Our findings offer an insight into microbiome changes during dispersal of a successful invader and present a baseline for assessment of their contribution to an invader’s overall health and its further invasion success.

**IMPORTANCE** Invasive species are among the major drivers of biodiversity loss and impairment of ecosystem services worldwide, but our understanding of their invasion success and dynamics still has many gaps. For instance, although it is known that host-associated microbial communities may significantly affect an individual’s health and fitness, the current studies on invasive species are mainly focused on pathogenic microbes, while the effects of the remaining majority of microbial communities on the invasion process are almost completely unexplored. We have analyzed the microbiome of one of the most successful crayfish invaders in Europe, the signal crayfish, and explored its changes along the signal crayfish invasion range in the Korana River, Croatia. Our study sets the perspective for future research required to assess the contribution of these changes to an individual’s overall health status and resilience of dispersing populations and their impact on invasion success.

## INTRODUCTION

The contribution of the microbiota in maintaining individual health and resilience of animal populations in the wild is being increasingly recognized (1) as well as its role in the context of biological invasions (2–5). Invasive alien species (IAS) are species that have been introduced either accidentally or intentionally outside their natural range and whose introduction and spread has negative effects on biodiversity, the economy, or human health in the new environment (6). They are recognized as a major driver of human-induced rapid environmental change (7) because they contribute to biodiversity loss, degradation of ecosystem structure, and impairment of ecosystem services worldwide (8, 9). Recent research demonstrated the effects of IAS on microbial communities in the novel environment. For example, biological invasions affect ecosystem functions, which may consequently drive changes in diversity and shifts in structure of environmental microbial populations (e.g., microbial diversity loss) (1, 5). Also, transmission of novel microbial pathogens is considered one of the main mechanisms through which IAS outcompete their native counterparts and pose a threat to human, animal, and ecosystem health (4, 10).

During the invasion process, successful invaders rapidly disperse within the novel environment (11), and microbes may play an important role in this process since microbial communities present in the novel environment, along with in the host’s microbiome, may affect host fitness (12). Although these interactions have been most frequently studied in the case of microbial pathogens, they apply to all microbes, because the effects of microbial community composition on host physiology, immune status, and overall fitness and health have been repeatedly demonstrated (1). For example, several studies suggest that during dispersal into the novel environment, an individual can lose its natural enemies (micropathogens), which may lead to lower prevalence of certain (i.e., pathogenic) microbial taxa in translocated populations of an invader or improve the condition of individuals at invasion fronts (a type of “enemy release”) (13–17). Furthermore, dispersing individuals may host microbes that are absent in the novel environment, which may lead to their establishment and spillover to the resident (native) species, giving the dispersing individuals a selective advantage in competition (spillover or novel weapon hypothesis) (18). Dispersing individuals can also acquire local microbes and serve as their reservoir, multiplying their (negative) impact on resident native species (spillback hypothesis) (19) but also with potential negative effects for the dispersing invader itself. Finally, microbial communities of the dispersing invader can contribute to the protection of their host by interfering with the entry of micropathogens into the host’s body and by preventing their establishment, growth, and spread (20). Therefore, both dispersal process and the characteristics of the novel environment may affect the structure and composition of a microbiome of a dispersing invader, which may indirectly and directly affect their health and their invasion success.

In this study, we analyzed the microbiome of a signal crayfish Pacifastacus leniusculus (Dana, 1852), the most successful crayfish invader in Europe, collected from a recently invaded Korana River in Croatia. We determined the differences between microbiomes of different tissues and examined changes in the microbiome along the signal crayfish invasion range. Invasive crayfish are one of the major threats to freshwater ecosystems (21) because they are among the most frequently translocated aquatic invertebrates that can dramatically modify freshwater communities and ecosystem functioning through combined impacts of consumption, competition, disease transmission, bioturbation, and mechanical destruction (21–24). Their introduction has been followed by rapid range expansion and a high number of documented negative impacts globally (25, 26). The North American signal crayfish is currently the most widespread crayfish invader in Europe, with records from 29 European countries (27) and is listed as a species of EU Concern according to the EU Regulation No. 1143/2014 on invasive alien species (6).

Signal crayfish were first recorded in the lower section of the Korana River in 2011 (28) and have been successfully spreading both upstream and downstream since (29, 30). Previous studies have recorded differences in signal crayfish population characteristics along its invasion range in the Korana River, with invasion fronts being male dominated and containing less aggressive individuals in better body and physiological condition (31, 32), which may be the result of nonrandom dispersal (33) and density-dependent effects. Given the observed differences between signal crayfish individuals from invasion core and invasion front, in this study, we explored whether such differences occur in its microbiome composition. We hypothesize that the composition of microbial communities of the signal crayfish is also affected by the range expansion and differs significantly along the invasion range. We aim to discern whether microbiomes are affected only by habitat characteristics when signal crayfish spread both upstream and downstream through the river or by a combination of nonrandom dispersal and density-dependent effects in an establishing population at the expanding edges.

To answer these questions, we have analyzed the microbiome of exoskeletal biofilm and multiple tissues (hemolymph, hepatopancreas, and intestine) of *P. leniusculus* using an amplicon sequencing approach based on the gene coding for 16S rRNA. We analyzed the above-mentioned microbiomes collected from four different locations along the signal crayfish invasion range in the Korana River and compared them to microbiomes present in the environment (water and sediment). Results of these analyses may help to understand the differences in microbiome composition of different tissues in crayfish and their changes during species dispersal through the novel environment.

## RESULTS

A total of 4,881,556 raw reads were obtained from the samples included in the study. After the DADA2 process (34) and filtering of the resulting feature table, 2,520,310 merged reads from 191 samples were obtained, and a total of 7,041 amplicon sequence variants (ASVs) were identified.

### Microbial diversity of crayfish-associated sample groups and the environment.

**FIG 1:**
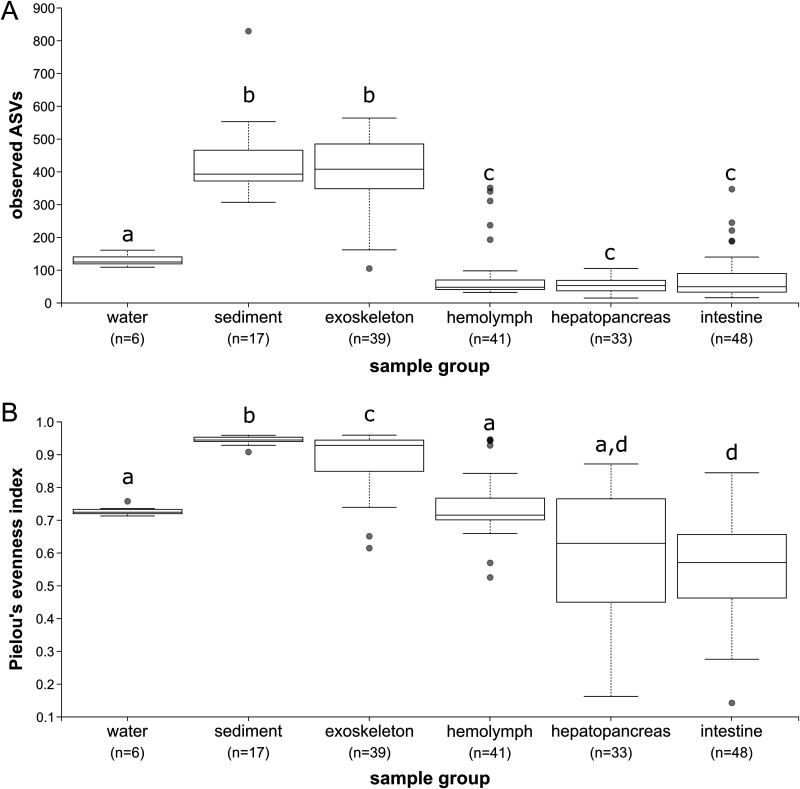
Alpha diversity analyses of microbiomes in different sample groups. (A) Observed ASVs. (B) Pielou’s evenness index. Significant differences are marked with different letters.

**(i) Alpha and beta diversity.** Overall, taxonomic richness (observed ASVs) and evenness (Pielou’s evenness index) differed significantly (Kruskal-Wallis test: *P* = 8.76E−24 and *P* = 1.03E−23, respectively) between six examined sample groups. Exoskeletal biofilm and sediment samples had the highest number of observed ASVs (Kruskal-Wallis test: *P* ≪ 0.01) compared to other sample groups, but no significant difference was recorded between the two (Fig. 1A). Similarly, these sample groups had a significantly higher evenness within a microbiome than other sample types, with significant differences between the sediment and the exoskeletal biofilm (*P* = 0.003) (Fig. 1B). Water samples differed significantly from all other 5 sample groups in the number of observed ASVs (*P* ≪ 0.01 in all cases) (Fig. 1A); however, water sample evenness was similar to that of the hepatopancreas and hemolymph (Fig. 1B). Three internal tissues (hemolymph, hepatopancreas, and intestine) exhibited no significant differences in richness or evenness except in the case of hemolymph, which had higher evenness than other internal tissues (Fig. 1B).

**FIG 2:**
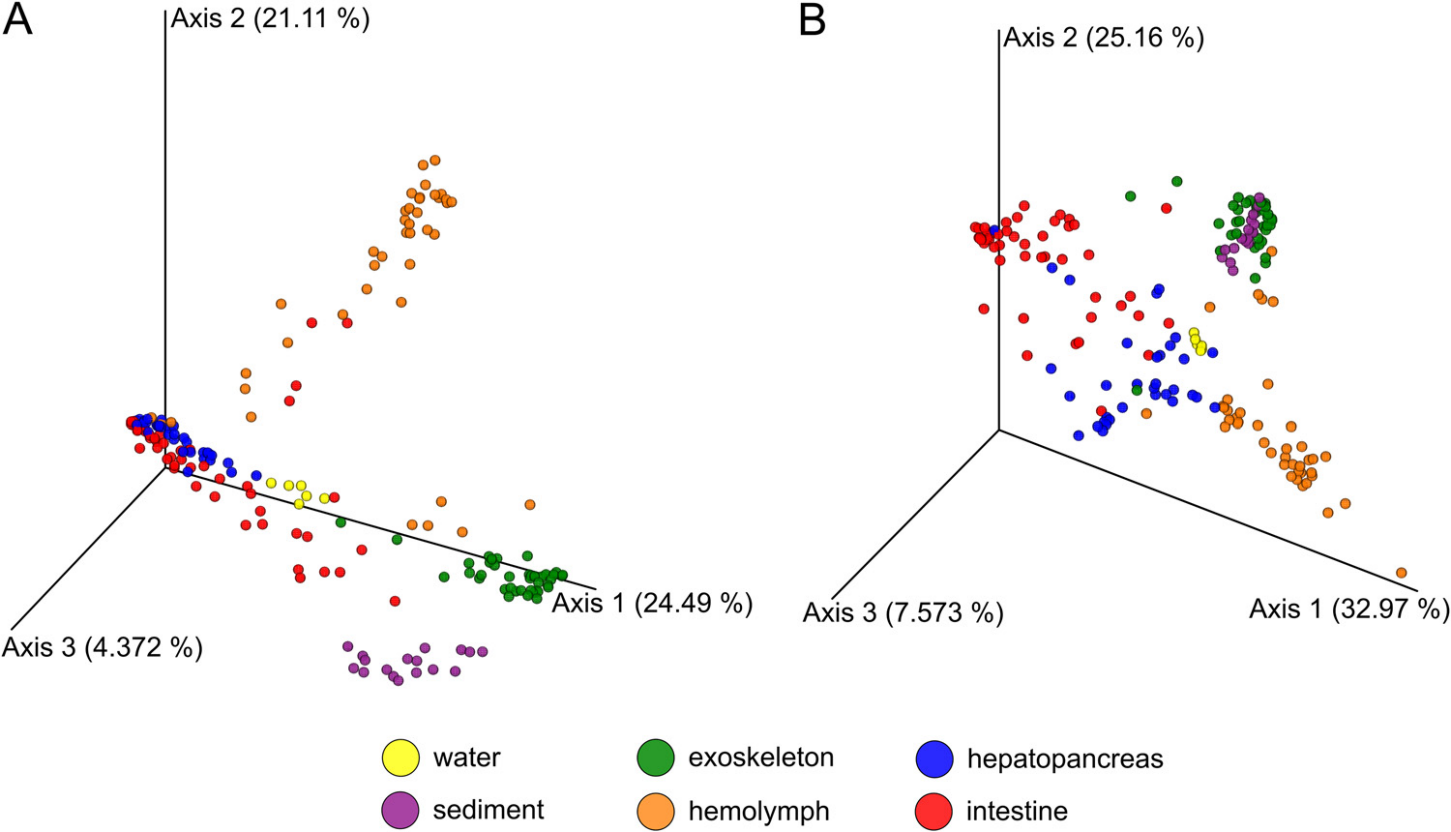
Beta diversity analyses of microbiomes between different sample groups. (A) Unweighted UniFrac. (B) Weighted UniFrac.

Both unweighted and weighted UniFrac showed an overall significant difference (permutational multivariate analysis of variance [PERMANOVA]: *P* = 0.001, pseudo-F = 26.3 and *P* = 0.001, pseudo-F = 50.5, respectively) between microbiomes of all six sample groups. Additionally, beta diversity pairwise tests showed a significant difference between all pairs of sample groups (*P* = 0.001). Intestine and hepatopancreas samples were grouped closely together in the unweighted UniFrac principal coordinates analysis (PCoA) (Fig. 2A) but not in the weighted UniFrac PCoA (Fig. 2B), while the opposite pattern was visible in the case of sediment and exoskeletal biofilm. In both analyses, hemolymph samples were positioned the farthest from other samples (Fig. 2A and B).

Additionally, comparisons of shared and unique ASVs between environmental samples (sediment, water) and each of the crayfish samples showed that the exoskeleton shared the highest number of ASVs with both water and sediment samples followed by the intestine (Fig. S1 in the supplemental material). Hepatopancreas and hemolymph shared the least ASVs with any of the environmental samples and were the sample groups with the highest percentage (70.5% hepatopancreas, 68.4% hemolymph) of unique ASVs compared to environmental samples. Compared to other crayfish samples, hepatopancreas had the lowest percentage of unique ASVs (Fig. S1E).

**FIG 3:**
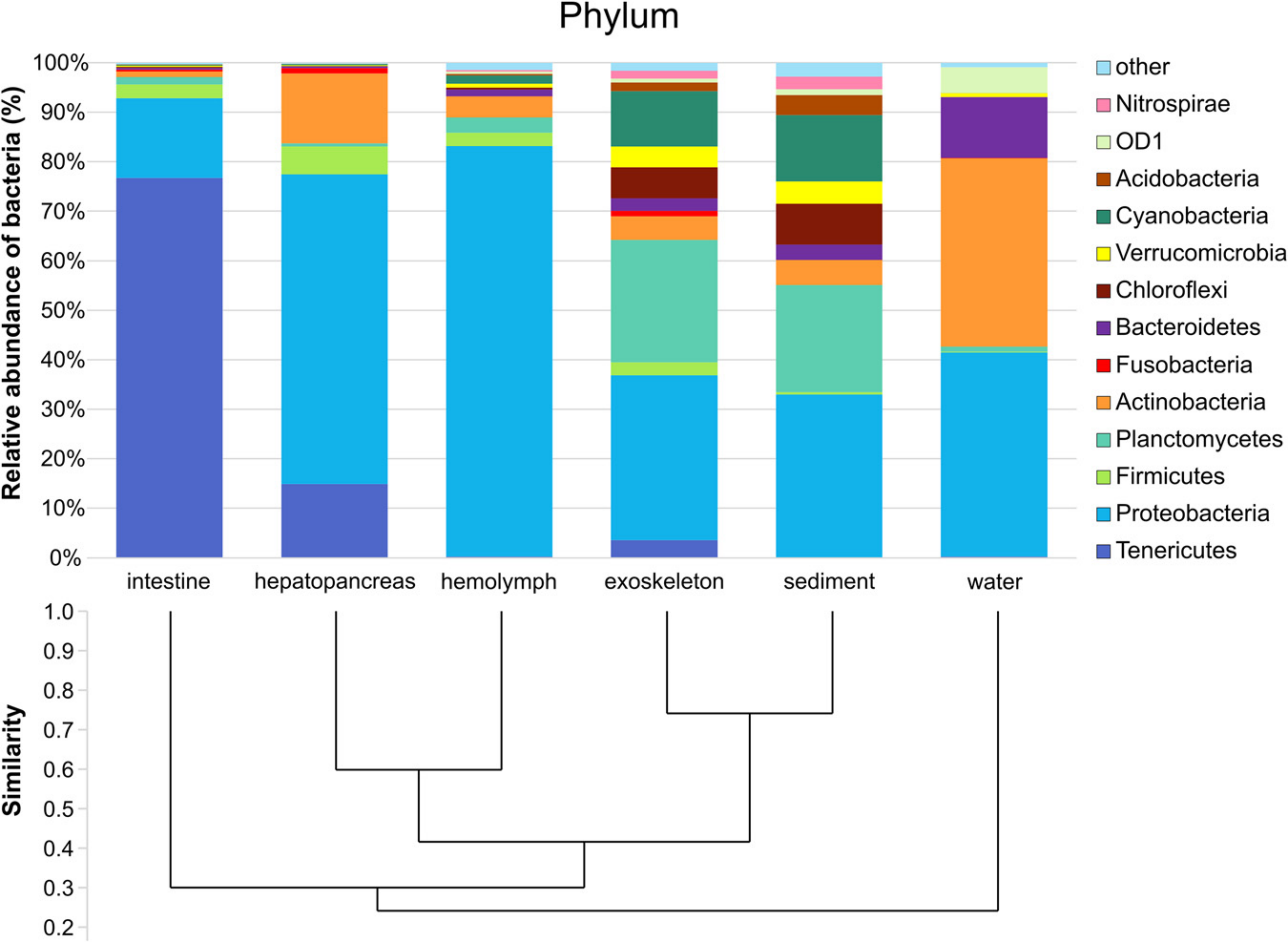
Relative abundance (%) of the overall most prevalent phyla and Bray-Curtis similarity index-based cluster analysis for all six sample groups. Bacterial phyla with an overall abundance of >1% are shown, while the remaining phyla were pooled and marked as “other.”

**FIG 4:**
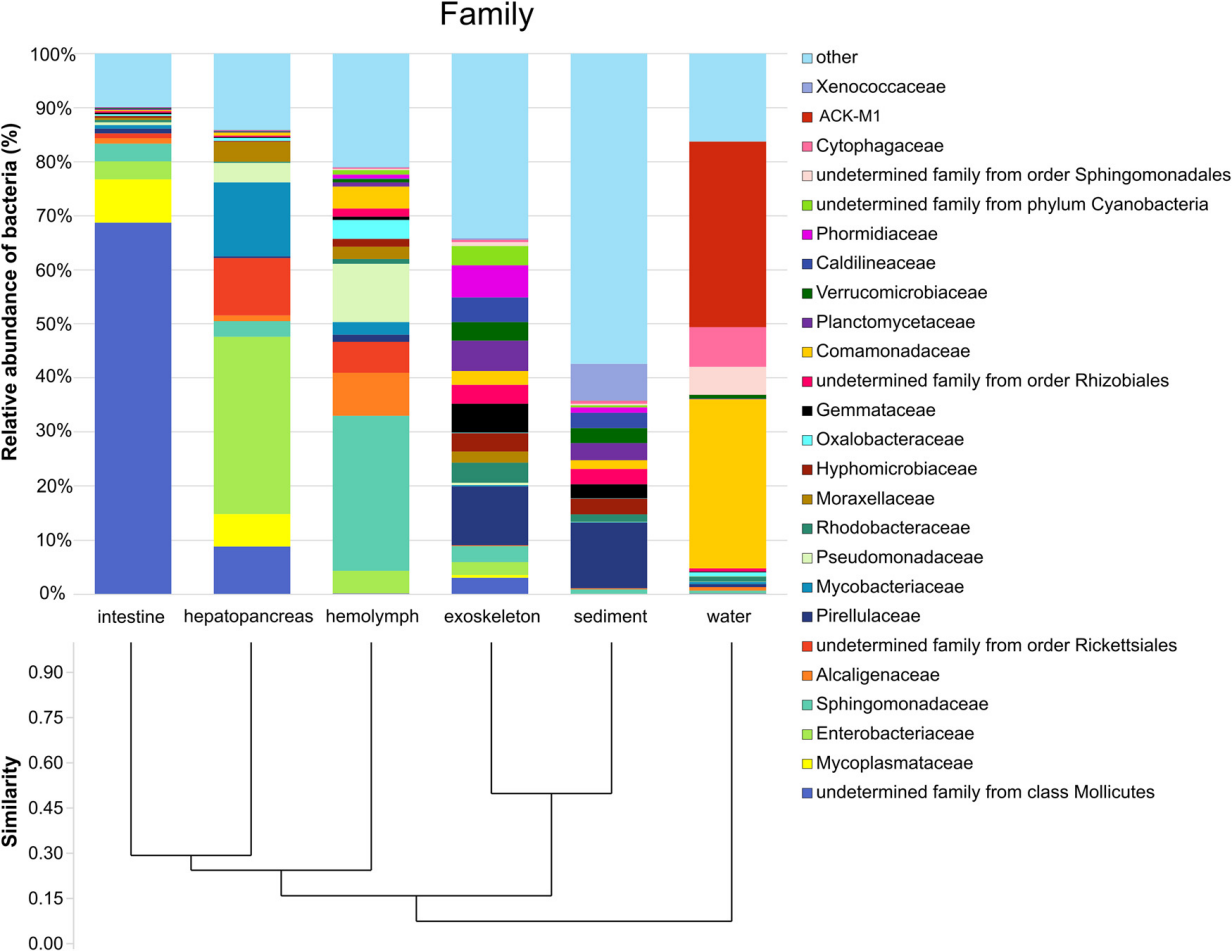
Relative abundance (%) of the overall most prevalent families and Bray-Curtis similarity index-based cluster analysis for all six sample groups. Bacterial families with an overall abundance of >3% are shown, while the remaining families were pooled and marked as “other.”

**(ii) Microbial composition.** At examined taxonomic levels, 49 phyla and 430 families were detected, with sediment and exoskeleton exhibiting similar composition and diversity patterns (Fig. 3 and 4). In addition to *Proteobacteria* (comprising 32.9% of sediment and 33.2% of exoskeletal microbiome), members of the phylum *Planctomycetes* represented 21.7% and 24.7% of the total community abundance in these two groups, with *Pirellulaceae* as the dominant family (Fig. 4). Unlike other sample groups, both sediment and exoskeleton showed notable abundances of the phylum *Cyanobacteria* (13.4% sediment and 11.2%, exoskeleton). Furthermore, sediment samples had the highest number of low-abundant families (54.7%) of all other sample groups (i.e., category ‘other’, families with abundance less than 3) (Fig. 4).

Intestine and hepatopancreas samples were dominated by the phyla *Tenericutes* (76.7% intestine and 14.9% hepatopancreas) and *Proteobacteria* (16.1% intestine and 62.8% hepatopancreas). At the family level, the intestine was dominated by an unknown family of *Mollicutes* class (68.7%) and the hepatopancreas by *Enterobacteriaceae* (32.8%), *Mycobacteriaceae* (13.6%), and an undetermined family of *Rickettsiales* order (10.6%) (Fig. 3 and 4). The hemolymph microbiome was dominated by members of the phylum *Proteobacteria* (82.9%), with *Sphingomonadaceae* (28.6%) as the dominant family followed by *Pseudomonadaceae* (10.8%). The water microbiome was dominated by *Proteobacteria* (41.3%, with dominant family *Comamonadaceae*) and *Actinobacteria* (38.1%, with dominant unnamed family ACK-M1), both of which were also ubiquitous in all sample groups. Additionally, water samples showed a relatively high abundance of bacteria belonging to the phylum *Bacteroidetes* (12.3%) in comparison to other sample groups, where it comprised 3.1% or less of the microbiome.

Finally, analysis of core features at the ASV level in all sample groups (sediment, exoskeleton, hemolymph, hepatopancreas, and intestine) at 90% sample inclusion identified the phylum *Proteobacteria* as the core feature in all of the sample groups, along with *Planctomycetes* (exoskeleton and sediment), *Verrucomicrobia* (sediment), and *Tenericutes* (intestine) (Table S1). Water samples had the most shared ASVs (47 core taxa at 100%; data not shown), while hepatopancreas had the smallest (0 core taxa at 100% and 1 core taxa only at 90%).

### Variation of the signal crayfish microbiome along its invasion range.

**FIG 5:**
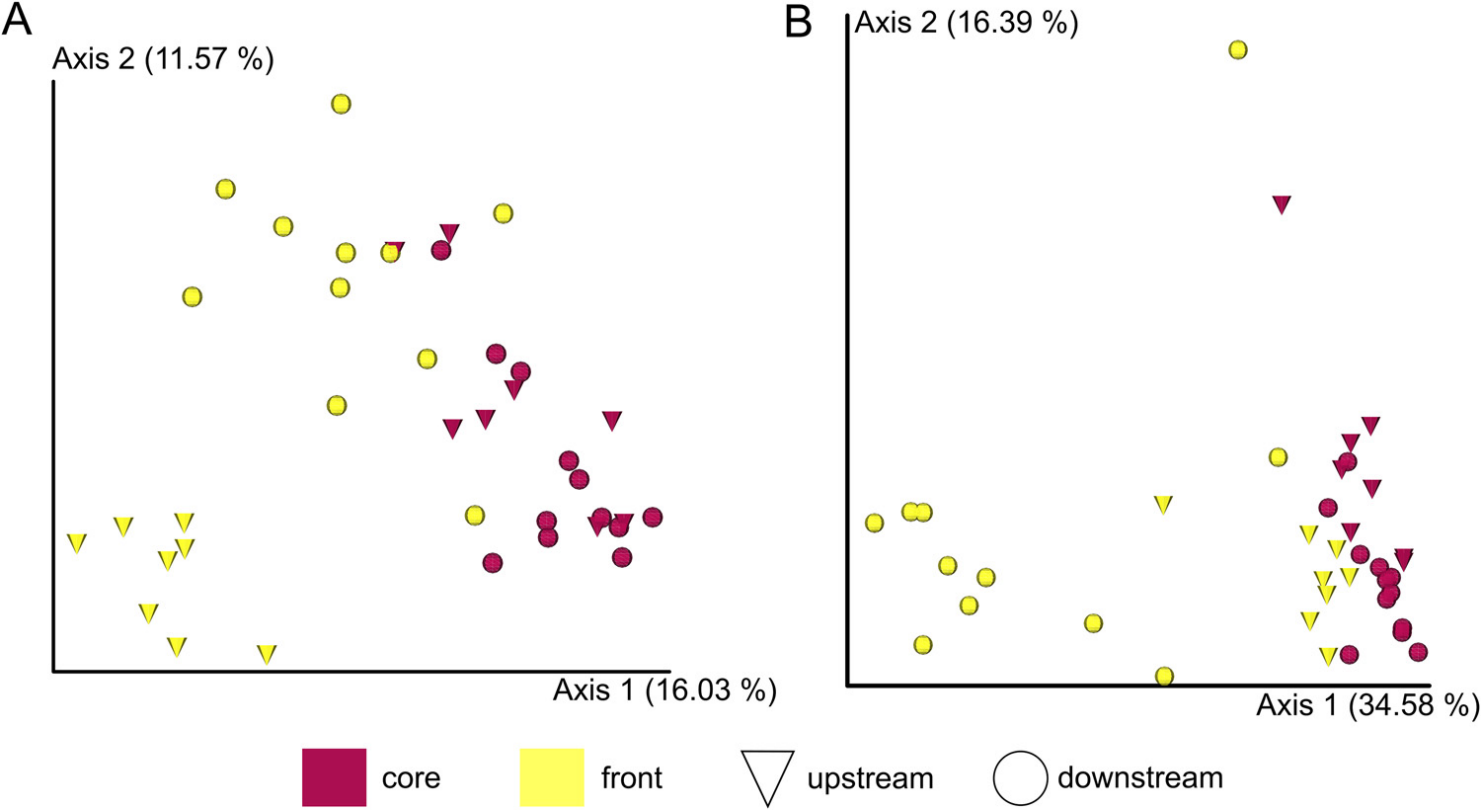
Beta-diversity metrics for the exoskeletal microbiome at all examined locations within the invasion range. Principal coordinate analysis (PCoA) is presented for unweighted (A) and weighted (B) UniFrac distances of individual crayfish.

Alpha diversity differed significantly only for hemolymph, with significant differences between downstream core and upstream sites (core and front) (Table S2 and Fig. S2). In beta diversity analyses, significant differences were observed for both unweighted (*P* = 0.001, pseudo-F = 4.072) and weighted UniFrac metrics (*P* = 0.001, pseudo-F = 9.076) for exoskeletal biofilm samples between all examined locations within the invasion range (upstream and downstream invasion fronts and upstream and downstream invasion cores) (Fig. 5A and B; Table S3). Sediment, intestine, and hemolymph samples did not exhibit any significant differences between examined locations (upstream invasion front [UF], upstream invasion core [UC], downstream invasion core [DC], and downstream invasion front [DF]) for unweighted and weighted UniFrac metrics; thus, they were pooled according to the invasion range (core versus front) and position in the river (upstream versus downstream part of the river). Sediment samples significantly differed between both the invasion range (core versus front) and position within the river (upstream or downstream part of the river) in both unweighted and weighted UniFrac analyses (Table S3).

The hemolymph microbiome differed significantly between upstream and downstream river sections for both unweighted and weighted UniFrac (*P* = 0.038, pseudo-F = 1.98 and *P* = 0.003, pseudo-F = 4.43, respectively) (Table S3) but not between invasion core and front. On the contrary, in intestine samples, a significant difference between the microbiomes of core and front populations was observed but only for unweighted UniFrac (*P* = 0.017, pseudo-F = 2.18) (Table S3), while no significant differences were observed between upstream and downstream segments of the river. A similar pattern was observed for hepatopancreas, where a significant difference was observed between the upstream invasion front and upstream core, but only in weighted UniFrac (*P* = 0.012, pseudo-F = 4.024), with no significant differences between upstream and downstream parts of the river.

Finally, significant differences in differential abundance of features were recorded in exoskeleton, hemolymph, and hepatopancreas but not in the intestine (Table S4). In the exoskeleton, *Cyanobacteria* and *Firmicutes* exhibited significant differences between examined locations, with the highest abundance of their genera Acinetobacter, *Macrococcus*, *Phormidium*, and *Aerococcus* at downstream invasion front (Table S4). In the case of hemolymph and hepatopancreas, genera *Caulobacter*, *Psychrobacter*, and Salmonella exhibited significant differences in abundance between examined locations (Table S4).

## DISCUSSION

In addition to negative effects on biodiversity, the economy, and human health, biological invasions may drive emergence of (new) diseases and changes in diversity and structure of microbial populations in the novel environment and may also affect population dynamics of invasive species (5, 35). Here, we analyzed differences in the microbiomes of different tissues of the successful freshwater invader the signal crayfish. Also, we examined whether differences in the microbiome occur during the invasion process and whether they are more pronounced along different river sections (downstream versus upstream; a proxy for the effect of microhabitat characteristics) or between crayfish populations of different density and species composition (core versus front; a proxy for the effect of changing population characteristics along the invasion range, that is, nonrandom dispersal and density-dependent effects). As effects of both resident and invader microbiota are increasingly recognized among the drivers of invasion success (35, 36), our results offer a baseline for better understanding their role and dynamics during range expansion.

### Composition and diversity of the bacterial communities associated with the signal crayfish.

Because there is little comparative information on the crayfish microbiome, except for several studies of single tissues (37–39), we analyzed microbial composition and diversity of different internal organs and tissues (hemolymph, hepatopancreas, and intestine) and exoskeleton and compared them to environmental samples (water and sediment). In all types of crayfish samples, *Proteobacteria* were the dominant phylum, which is consistent with previous research on other crustaceans (40–43) and other aquatic invertebrates (44). This indicates that this phylum is important for the host and ubiquitous in the environment. Out of all analyzed samples, sediment and exoskeletal microbiomes were the most taxonomically rich and uniform. The identified dominant family *Pirellulaceae* in the exoskeleton and sediment in our study was also among the most represented in other crayfish species (i.e., *Cambarus sciotensis*) (37). Also, exoskeleton samples shared the highest percentage of ASVs with both the sediment and water samples. This is not surprising since the exoskeleton is simultaneously a barrier and a link between crayfish and the environment, and crayfish are in continuous contact with the sediment (bioturbators) (45, 46) during their life span. However, beta diversity analyses showed significant differences between sediment and the exoskeleton, despite their close grouping in weighted UniFrac. Thus, in addition to the characteristics of rich and diverse bacterial communities in the sediment, which were shown to shape the crayfish exoskeletal microbiome (37) and skin microbiome of other aquatic species (i.e., fish [47–50]), other factors such as exoskeleton characteristics (i.e., cuticle morphology and structure, presence of microinjuries, or time since last molt) and population characteristics (i.e., density, structure, number of species present, and their physiological status, which is discussed later in the text) may also influence the microbiome composition.

Internal tissues (hemolymph, hepatopancreas, and intestine) were significantly less rich in ASVs and exhibited lower evenness. Hemolymph had the lowest (albeit not significant) richness out of all internal tissues. While microbial communities in hemolymph are generally considered less rich and abundant than other organs due to its regulation by the host immune response (51, 52), this study, along with some other studies (52), demonstrated similarity in microbial community richness of hemolymph and the hepatopancreas. In addition to harboring potentially pathogenic microbes or opportunistic micropathogens, hemolymph may also contain symbiotic microbes that may help boost the host’s immune response or even inhibit pathogen proliferation (52). Beta diversity analyses showed that hemolymph had the most distinct microbiome composition and feature abundance compared to other analyzed crayfish or environmental samples. This could be explained by its specificity compared to other crayfish sample groups (circulating liquid tissue with many antimicrobial components tightly controlled by the host’s immune system [51–53]). However, the fact that hemolymph is in direct contact with all internal organs (54) explains the observed significant portion (>80%) of shared ASVs with other crayfish tissues. Additionally, some studies (52, 55) support the hypothesis that microbes may be translocated from the digestive tract (hepatopancreas and intestine) to hemolymph in invertebrates with open circulatory systems.

Intestine samples had the lowest evenness of all samples, since certain ASVs (i.e., members of the class *Mollicutes*) dominated the intestinal microflora. Also, intestine and hepatopancreas samples, albeit being significantly different, shared similar ASVs (unweighted UniFrac analyses) but in different abundances (weighted UniFrac analyses). Intestinal and hepatopancreatic communities are probably partly determined by the type of food since they are both parts of the digestive system, and multiple studies highlight diet as one of the main drivers in shaping the host’s intestinal microbiome (38, 56–58). However, the functions of these organs differ significantly, which may explain differences in abundance of shared features, as hepatopancreas is a digestive gland involved in metabolism and absorption of low-molecular-weight nutrients, while the intestine plays a role in digestion, ion osmoregulation, and water uptake (54). The latter is visible from the high number of shared ASVs between the intestine and sediment and water samples observed in this study.

### Variation of the signal crayfish microbiome along its invasion range.

In concordance with our hypothesis, the crayfish microbiome demonstrated differences along the invasion range; the analyzed signal crayfish samples exhibited both variation in respect to their position along the invasion range (invasion core versus invasion front: exoskeleton, hepatopancreas, and intestine) as well as in respect to their position along the river (upstream or downstream section of the river: exoskeleton and hemolymph). The exoskeletal microbiome varied significantly between all examined locations, while sediment samples exhibited a similar pattern of variation in beta diversity between the core and front as well as upstream and downstream parts of the river. This, in addition to the comparative analyses of alpha diversity of sediment and exoskeletal samples, corroborates that the exoskeletal microbiome is shaped to a high extent by local environmental characteristics, as recorded in previous studies (37). However, the significant difference in diversity and abundance of sediment and exoskeleton samples (discussed in chapter above) indicates that other factors besides the characteristics of the local environment affect the exoskeletal microbiome composition. As the exoskeletal microbiome is determined by the available regional microbial species pool, which also includes microbes of all host individuals in a given environment (59), we suggest that crayfish density and population structure may significantly affect its composition and lead to the observed high variation in diversity and abundance among all locations and contribute to the observed significant differences from the environmental (sediment) microbiome. In the invasion core populations, only signal crayfish are present in high abundance, while invasion fronts have 7 to 8 times lower signal crayfish abundance than invasion cores and also cooccur with the native congener *Pontastacus leptodactylus* (Eschscholtz, 1823) (30). Aggression plays an important role in the dynamics of crayfish populations (60), and high crayfish abundance has been shown to increase the competition for limited resources and the rate of interaction between individuals (61). Thus, as contact rates between individuals increase with increasing density, this may also elevate the transmission of microbiota between individuals, as established in the case of pathogens (62, 63).

Hemolymph exhibited significant differences in the microbiome composition and feature abundance between upstream and downstream river sections, but not in respect to the position along the invasion range (invasion core versus front). Under favorable conditions, homeostasis exists between the microbial communities of the hemolymph and the host (51), with the composition and abundance of bacterial communities remaining relatively stable (64). However, under stress, significant community changes may occur since many hemolymph bacteria are opportunistic pathogens that may proliferate under stressful conditions, induce bacterial septicemia, and adversely affect crayfish health (42, 65). Because multiple factors (i.e., changes in the environment, host physiology status, microbe-microbe interactions in a tissue, etc.) (11, 59, 66, 67) can lead to changes in the microbiome, the observed differences in the feature abundance and composition of the hemolymph microbiome at the upstream and downstream locations may be driven by differences in characteristics of these two environments, as the upstream section of the studied area of the Korana River flows through the sparsely populated rural region, while the downstream section of the river passes through the industrial zone of the Karlovac city. Future studies should address the observed changes in the hemolymph microbiome along with detailed analyses of the water quality parameters and crayfish immune response at each site to address this question.

The analyses of intestinal and hepatopancreatic microbiomes (unweighted and weighted UniFrac tests) showed significant differences between core and front populations. Hepatopancreatic microbiomes were composed mostly of the same taxa, but with different abundances at (upstream) the invasion core and front. On the contrary, intestinal microbiomes exhibited significant differences in taxonomic composition between invasion core and front populations but had similar abundances. We hypothesize that observed differences in both abundance in the hepatopancreas and diversity of taxa in the intestine are dependent on the crayfish feeding regimen and crayfish condition and driven by both environmental conditions (i.e., type of available food) and density-dependent effects. Crayfish are omnivorous and ingest large amounts of detrital materials during feeding (68), which is also visible from the high number of shared ASVs between sediment and the intestine. In this study, we identified differences in sediment microbiome composition and abundance along the invasion range and river sections, which indicates the potentially different composition of detrital materials ingested between the sites. Also, as already discussed, examined populations at invasion cores and fronts differed significantly in crayfish density. In the latter case, the higher intensity of resource competition in highly abundant populations at invasion cores may affect crayfish diet (i.e., feeding rates, patterns, and preferred food availability) (61). The latter may have a more pronounced impact on the observed differences in abundance of particular groups of intestinal microbes between invasion core and front populations than the composition of detrital material ingested. Additionally, previous studies have demonstrated the link between individual fitness and the composition of the intestinal microbiota (reviewed in reference 1), while studies on the signal crayfish in the Korana River demonstrated that its condition (measured using hepatosomatic and body condition indices) was lower in the core than in the invasion fronts (32). Inferior crayfish condition along with the potentially limited variety and abundance of food sources (69, 70) at invasion cores may thus affect the observed differences in the intestinal microbiome composition between invasion core and invasion front populations (higher observed diversity at invasion fronts). The impact of an animal’s condition on the composition and diversity of the gut microbiota has been corroborated in crayfish (71, 72) as well as other aquatic animals (i.e., shrimps [73–75] and fish [76, 77]). Crayfish condition (i.e., its density dependence) may also be driving the observed changes in hepatopancreas community composition along the invasion range. In crustaceans, the hepatopancreas plays an important role in lipid metabolism as a main energy storage organ that supports key physiological functions, such as reproduction, movement, and growth (54, 78, 79). Due to its role in food degradation and due to its specific organ environment (i.e., low pH, presence of digestive enzymes) (80), it may filter which bacteria will successfully colonize it. This may explain the similarity in the composition of the microbiomes of both core and front populations. Also, in other decapods, the hepatopancreas has been shown to have a more conserved and distinct microbiome than the intestine (41, 74), which could explain that differences were observed in the feature abundance, but not in the microbiome composition, along the invasion range. Further studies involving *in situ* research, behavioral studies, analyses of crayfish condition, diet (stable isotope analyses), and the subsequent analyses of the hepatopancreas and intestinal microbiota are required to link more precisely crayfish diet and density to the individual’s relative condition, nutrient assimilation patterns, and gastrointestinal and hepatopancreatic microbiome changes, similar to the studies performed in fish (81).

Finally, we expected to observe differences in abundance of some genera, which include crayfish micropathogenic taxa (82), based on suggested hypotheses of their effect during invader’s dispersal (i.e., enemy release, spillover effect, spill back effect) (14, 18, 19). However, genera for which the differences were observed along the invasion range were not among the genera with crayfish micropathogens for the specific tissues known from the literature (82) and were not considered pathogenic or classified as potential micropathogens in other aquatic species (i.e., *Psychrobacter*) (83–86). To further investigate the effect of micropathogens on invasion dynamics of the signal crayfish in the Korana River, targeted monitoring of well-established crayfish micropathogens for which the detection assays are available (i.e., *Aphanomyces astaci*, infectious hematopoietic necrosis virus, *Macrobrachium rosenbergii* nodavirus (MrNV), and white spot syndrome virus [82, 87]) is required. Also, since crayfish diseases are still largely under researched (82), further studies into the potentially pathogenic microbial species in crayfish from the genera identified within this study are needed. This is especially relevant in the case of the hepatopancreas, in which acute idiopathic necrotic hepatopancreatitis has been observed extensively in the signal crayfish from the Korana River and which exhibits significant differences in its incidence and severity along the invasion range (88).

In conclusion, we demonstrated differences in the composition of the signal crayfish microbiome along its invasion range, suggesting that microbial communities may affect and be affected by range expansion. Thus, our study sets the perspective for future research required to assess the contribution of the changes in the microbiome to an individual’s overall health status, resilience of dispersing populations, and their invasion success. However, microbiomes associated with different crayfish species and organs are currently largely unknown, and detailed studies are needed to describe the microbiome of healthy animals, which could then be used to detect deviations that could be linked with biotic and abiotic environmental stressors often at play during biological invasions.

## MATERIALS AND METHODS

### Study area.

**FIG 6:**
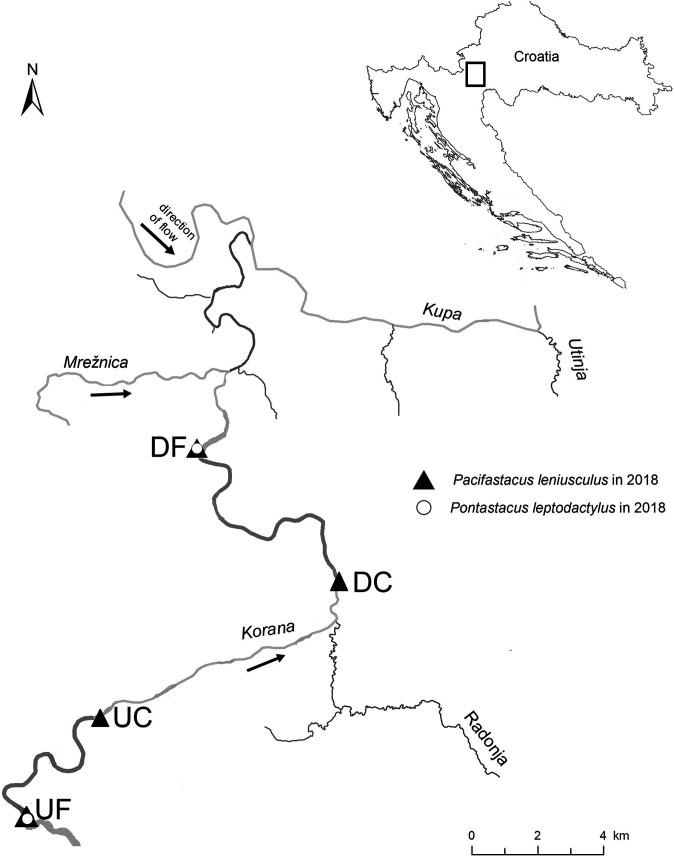
Position of sampling sites along the invasion range of the signal crayfish in the Korana River in 2018. Sampling was performed at both upstream (UF) and downstream (DF) invasion fronts and upstream (UC) and downstream (DC) invasion cores.

We sampled the lower reaches of the Korana River, a 134-km-long karstic river located in continental Croatia that belongs to the Sava River basin, where signal crayfish are spreading both upstream and downstream (30). The upstream section of the studied area flows through the sparsely populated rural region, while in the downstream section, the river passes through the industrial zone and flows into the Kupa River in the city of Karlovac. Along the whole length of its course, multiple natural and human-made cascades are present (29). The study area includes sites differing in crayfish community composition (i.e., dense intraspecific populations of *P. leniusculus* and less abundant heterospecific populations of *P. leniusculus* and *P. leptodactylus*) (Fig. 6).

### Sampling procedure.

Fieldwork was conducted during the period of increased crayfish activity of both sexes (i.e., before the mating period [89]) in the early autumn of 2018. The ongoing context of range expansion allowed us to sample signal crayfish individuals from two distributional ends: (i) invasion core (longer established population with higher crayfish abundance) and (ii) invasion front (recently established population at the edge of the range with lower crayfish abundance). The crayfish were captured at four sites along the 33 km of the Korana lower watercourse, which were previously (30) identified as upstream invasion front (UF), upstream invasion core (UC), downstream invasion core (DC), and downstream invasion front (DF) (Fig. 6). Upstream and downstream invasion cores have 7 to 8 times higher relative *P. leniusculus* abundance than invasion fronts and contain no native crayfish since they were outcompeted from these sites, while at invasion fronts, *P. leniusculus* cooccurs in interspecific populations with the native *P. leptodactylus* (29, 30).

Crayfish were captured using baited LiNi traps (90), which were left in the water overnight. Following capture, individuals were identified to species level by visual inspection. Captured native *P. leptodactylus* were returned to the river, while a total of 110 *P. leniusculus* individuals of both sexes (27 from UF, 23 from DF, 30 from UC, and 30 from DC) were placed in individual containers on ice and taken to the laboratory for tissue sample collection. Additionally, environmental samples were collected at all sites; water was sampled using 1,000-ml sterile bottles (one bottle at each invasion core, two bottles at invasion fronts), and sediment was taken as composite samples (4 to 5 samples at each site, which were collected approximately 1 to 2 m apart, from the surface of the sediment [0 to 5 cm]) using a sterile sampling spoon and immediately transported to the laboratory on ice.

In the laboratory, collected water samples were vacuum filtered through 0.22-μm pore-size membrane (MCE) filters, which were stored at −20°C until DNA extraction. Four types of crayfish samples were taken for each individual crayfish: exoskeletal biofilm, hemolymph, hepatopancreas, and intestine (i.e., midgut and hindgut). Exoskeletal biofilm was sampled by taking cuticle swabs as previously described (91). Briefly, any loosely adhered debris (such as vegetation, mud, or sediment) was manually removed from the crayfish, which were then thoroughly scrubbed with a sterile brush wetted with a 0.1% NaCl and 0.15 M Tween 20 solution. After centrifugation of the suspension at 10,000 × *g* for 15 min at 4°C, the supernatant was discarded, and the pellet of epibiotic cells was frozen at −20°C. Next, we collected 400 μl of hemolymph in 200 μl of anticoagulant solution (0.49 M NaCl, 30 mM trisodium citrate, 10 mM EDTA) from the base of the individual’s walking leg (previously rinsed by 70% ethanol) by using a sterile needle as previously described (42). The collected hemolymph was centrifuged at 10,000 × *g* for 10 min at 4°C, and the pellet was frozen at −20°C until DNA extraction. For dissected organs (hepatopancreas and intestine), the sampling procedure was the same; the complete organ was removed from the body, placed in a sterile petri dish, and carefully chopped into small pieces using a sterile scalpel and frozen at −20°C. Nondisposable dissecting scissors were alcohol flame sterilized between each individual sample.

### DNA extraction.

Genomic DNA was extracted from exoskeletal biofilm, hemolymph, hepatopancreas, and intestine using the NucleoSpin microbial DNA kit (Macherey-Nagel, Germany) as per the manufacturer’s protocol for Gram-positive and Gram-negative bacteria and with modifications regarding sample lysis by agitation as previously described (91). Genomic DNA from sediment and water samples was extracted using a DNeasy PowerSoil Pro kit (Qiagen, Germany). Three replicates of each composite sediment sample were isolated from invasion cores and six from invasion fronts. To select the samples of highest quality for subsequent Illumina sequencing, we have analyzed the yield of metagenomic DNA samples and also tested the samples for the presence of bacterial DNA. DNA quantity was analyzed in all samples using the QuantiFluor ONE double-stranded DNA (dsDNA) system and the Quantus Fluorometer (Promega, USA). Further, the presence of bacterial DNA in the samples was confirmed by PCR; that is, almost full-length 16S rRNA gene amplification (using primers 27F and 1492R as previously described [92]) was conducted on all samples. Finally, we chose 192 samples from all six sample groups for amplicon sequencing of variable regions 3 and 4 of the 16S rRNA (Table S5 in the supplemental material) based on the following criteria: (i) satisfactory DNA concentration, (ii) successful 27F/1492R PCR amplification of the 16S rRNA gene, and (iii) relatively uniform coverage of different sampling locations.

### Library preparation, sequencing, and bioinformatics analysis.

Amplification and sequencing of the variable V3-V4 region of the 16S rRNA gene was performed by Microsynth, Switzerland. An Illumina library was prepared using 16S Nextera two-step PCR using forward 341F (5′-CCTACGGGNGGCWGCAG-3′) and reverse 802R (5′-GACTACHVGGGTATCTAATCC-3′) primers and sequenced on an Illumina MiSeq using the MiSeq reagent kit v2 (2 × 250 bp paired-end). Illumina raw paired-end sequences were analyzed in ‘Quantitative Insights Into Microbial Ecology 2’ (QIIME2) software (93), release 2019.10. Raw demultiplexed paired-end fastq files were imported into QIIME2 using a manifest file and were then quality filtered, trimmed, dereplicated, denoised, merged, and assessed for chimaeras to produce ASVs using the DADA2 plugin (34). The DADA2-generated feature table was filtered to remove ASVs at a frequency of less than 10 per sample and appearing in less than two samples. Taxonomy was assigned to ASVs using a pretrained naive Bayes classifier. The classifier was trained on the Greengenes 13_8 99% operational taxonomic unit (OTU) data set, targeting the V3 and V4 region of the 16S rRNA gene using the QIIME2 feature classifier plugin (94). Based on the generated taxonomy, the feature table was filtered to exclude ASVs assigned to the class *Chloroplast*. A phylogenetic tree was generated using fasttree2 based on mafft alignment of ASVs as implemented in the q2-phylogeny plugin. The microbial diversity and richness of all samples were estimated using alpha (Pielou’s evenness index and observed ASVs) and beta (unweighted and weighted UniFrac (95) diversity metrics using the diversity plugin within QIIME2. Alpha and beta diversity metrics were calculated for (i) all six groups of samples with the samples of the same type pooled across sites (to analyze the microbial diversity of crayfish tissues and environment; subsampled to 1,402 reads per sample; 184 samples in total) (Table S5A) and (ii) each group of tissue samples separately between four sites (to analyze differences in composition of the microbiome along the invasion range; subsampled to 2,464 reads per sample for exoskeletal biofilm, 2,285 for hemolymph, 1,883 for hepatopancreas, 6,603 for intestine, and 4,909 for sediment; 173 samples in total) (Table S5B). UniFrac diversity metrics were visualized by generating a principal coordinates analysis (PCoA) plot using Emperor (96). Differences along the invasion range and between different river segments were tested with Benjamini-Hochberg corrected Kruskal-Wallis and PERMANOVA tests (97) for alpha and beta diversity, respectively. Since no significant differences between sexes were established for any of the crayfish sample groups (exoskeletal biofilm, hemolymph, hepatopancreas, and intestine) in both alpha and beta diversity analyses, both sexes were pooled. Furthermore, if no significant differences were observed between upstream and downstream invasion fronts or upstream and downstream invasion cores, they were pooled into either the front or core group (differences along the invasion range) and upstream or downstream group (differences between river segments). Additionally, we determined shared and unique ASVs between environmental samples and each analyzed crayfish sample groups as well as between all crayfish samples by using Venn diagrams, which were visualized using an online tool (http://bioinformatics.psb.ugent.be/webtools/Venn/). Analysis of compositions of microbiomes (ANCOM) tests (98) were used to identify ASVs that are differentially abundant between locations using the composition plugin within QIIME2. Further analysis was performed to establish core features present in high numbers of samples using the feature-table QIIME2 plugin (99). Finally, to reveal the similarities in microbial composition on phylum and family levels, the Bray-Curtis similarity index-based cluster analysis was performed using PAST software (100).

### Data availability.

The next-generation sequencing data that support the findings of this study are openly available in the EMBL Nucleotide Sequence Data Base (ENA) at https://www.ebi.ac.uk/ena/browser/home, reference number PRJEB43749.
