# Supplementary material for: Microbiome of the Successful Freshwater Invader, the Signal Crayfish, and Its Changes along the Invasion Range
Source: Microbiol Spectr. 2021 Sep 8;9(2):e00389-21. doi: 10.1128/Spectrum.00389-21 (PMC8557874; doi:10.1128/Spectrum.00389-21)
Supplement: SUPPLEMENTAL FILE 1 — Supplemental material. Download SPECTRUM00389-21_Supp_1_seq4.docx, DOCX file, 0.3 MB [file spectrum00389-21_supp_1_seq4.docx]

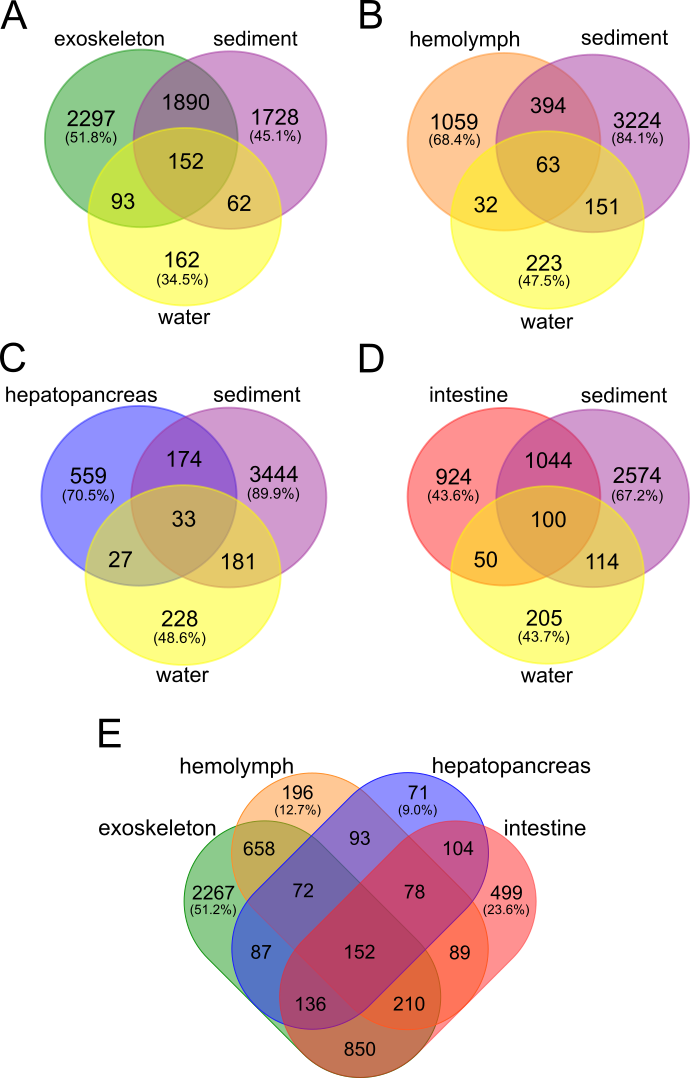


**FIG. S1.** Venn diagrams showing the numbers of shared and unique ASVs between sediment and water samples, and exoskeletal biofilm (A), hemolymph (B), hepatopancreatic (C) and intestinal (D) samples. The numbers of shared and unique ASVs between four groups of crayfish samples (exoskeletal biofilm, hemolymph, hepatopancreatic, intestinal) are visualized in E.

**TABLE S1.** Core features at ASV level in sediment, exoskeleton, hemolymph, hepatopancreas and intestine at 90 % sample inclusion.

| SAMPLE GROUP | CORE FEATURES (at 90% sample inclusion) |
| --- | --- |
| exoskeleton | k__Bacteria; p__Proteobacteria; c__Alphaproteobacteria; o__Rhodobacterales; f__Rhodobacteraceae; g__Rhodobacter; s__ |
|  | k__Bacteria; p__Proteobacteria; c__Alphaproteobacteria; o__Sphingomonadales; f__; g__; s__ |
|  | k__Bacteria; p__Proteobacteria; c__Alphaproteobacteria; o__Rhodobacterales; f__Rhodobacteraceae; g__Rhodobacter; s__ |
|  | k__Bacteria; p__Planctomycetes; c__Phycisphaerae; o__WD2101; f__; g__; s__ |
|  | k__Bacteria; p__Planctomycetes; c__Planctomycetia; o__Pirellulales; f__Pirellulaceae; g__; s__ |
|  | k__Bacteria; p__Planctomycetes; c__Planctomycetia; o__Pirellulales; f__Pirellulaceae; g__; s__ |
|  | k__Bacteria; p__Planctomycetes; c__Planctomycetia; o__Pirellulales; f__Pirellulaceae; g__; s__ |
| hemolymph | k__Bacteria; p__Proteobacteria; c__Alphaproteobacteria; o__Sphingomonadales; f__Sphingomonadaceae; g__Sphingomonas; s__ |
|  | k__Bacteria; p__Proteobacteria; c__Alphaproteobacteria; o__Sphingomonadales; f__Sphingomonadaceae; g__Sphingomonas; s__ |
|  | k__Bacteria; p__Proteobacteria; c__Betaproteobacteria; o__Burkholderiales; f__Alcaligenaceae; g__Achromobacter; s__ |
|  | k__Bacteria; p__Proteobacteria; c__Gammaproteobacteria; o__Pseudomonadales; f__Pseudomonadaceae; g__Pseudomonas; s__ |
|  | k__Bacteria; p__Proteobacteria; c__Betaproteobacteria; o__Burkholderiales; f__Oxalobacteraceae; g__Cupriavidus; s__ |
|  | k__Bacteria; p__Proteobacteria; c__Betaproteobacteria; o__Burkholderiales; f__Burkholderiaceae; g__Burkholderia; s__ |
|  | k__Bacteria; p__Proteobacteria; c__Gammaproteobacteria; o__Pseudomonadales; f__Pseudomonadaceae; g__Pseudomonas; s__ |
|  | k__Bacteria; p__Proteobacteria; c__Betaproteobacteria; o__Burkholderiales; f__Comamonadaceae |
|  | k__Bacteria; p__Proteobacteria; c__Gammaproteobacteria; o__Enterobacteriales; f__Enterobacteriaceae; g__Gluconacetobacter; s__ |
| hepatopancreas | k__Bacteria; p__Proteobacteria; c__Gammaproteobacteria; o__Pseudomonadales; f__Pseudomonadaceae; g__Pseudomonas; s__ |
| intestine | k__Bacteria; p__Tenericutes; c__Mollicutes; o__; f__; g__; s__ |
|  | k__Bacteria; p__Tenericutes; c__Mollicutes; o__; f__; g__; s__ |
|  | k__Bacteria; p__Proteobacteria; c__Alphaproteobacteria; o__Sphingomonadales; f__Sphingomonadaceae; g__Sphingomonas; s__ |
|  | k__Bacteria; p__Proteobacteria; c__Betaproteobacteria; o__Burkholderiales; f__Alcaligenaceae; g__Achromobacter; s__ |
| sediment | k__Bacteria; p__Planctomycetes; c__Planctomycetia; o__Pirellulales; f__Pirellulaceae; g__Pirellula; s__ |
|  | k__Bacteria; p__Proteobacteria; c__Gammaproteobacteria; o__Xanthomonadales; f__Sinobacteraceae; g__; s__ |
|  | k__Bacteria; p__Verrucomicrobia; c__Verrucomicrobiae; o__Verrucomicrobiales; f__Verrucomicrobiaceae; g__Luteolibacter; s__ |
|  | k__Bacteria; p__Planctomycetes; c__Planctomycetia; o__Pirellulales; f__Pirellulaceae; g__; s__ |
|  | k__Bacteria; p__Proteobacteria; c__Alphaproteobacteria; o__Rhizobiales; f__Hyphomicrobiaceae; g__; s__ |

**TABLE S2**. Results of Kruskal-Wallis test of differences in alpha diversity of observed ASVs between crayfish populations (core-front) and locations (upstream-downstream), with Benjamini-Hochberg FDR correction of P-values. If locations (UF, UC, DC, DF) exhibited significant differences (as for hemolymph samples), they were analyzed separately, if not they were pooled together. Statistically significant results are marked in red.

| **OBSERVED ASVs** | | | |
| --- | --- | --- | --- |
| **sample** | **p values: core-front** | | **p values: upstream-downstream** |
| exoskeleton | 0.715 | | 0.475 |
| hepatopancreas | 0.771 | | 0.101 |
| intestine | 0.059 | | 0.519 |
| sediment | 1.0 | | 0.149 |
| **sample** | **locations** | | **p values** |
| hemolymph | downstream core (n= 11) | upstream core (n = 7) | 0.031 |
|  |  | upstream front (n = 11) | 0.031 |


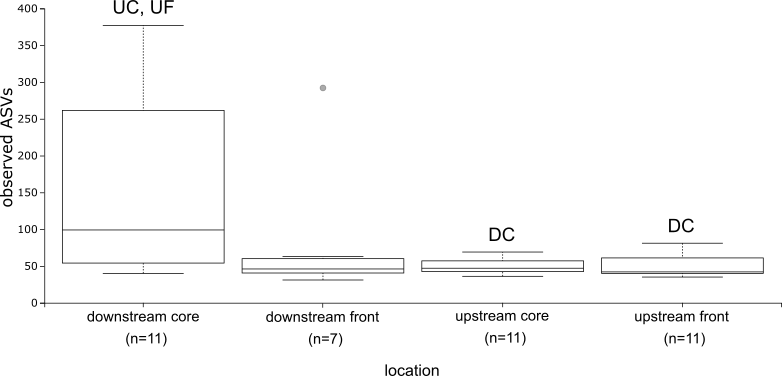


**FIG. S2.** Alpha diversity analyses of hemolymph microbial communities along invasion range. Downstream core exhibited significantly higher number of observed ASVs in comparison to upstream core and front sites. Significant differences between specific groups were marked with their abbreviations (UC = upstream core, DC = downstream core, UF = upstream front).

**TABLE S3.** Beta diversity analyses along the invasion range. Samples that differed significantly between locations (UF, UC, DC and DF) are shown in the lower part of the table, while others are grouped by position in the invasion range (core and front) and river section (upstream and downstream). DC= downstream core, DF = downstream front, UC = upstream core, UF = upstream front. Statistically significant results are marked in red.

| **sample group** | **unweighted UniFrac** | | | | **weighted UniFrac** | | | |
| --- | --- | --- | --- | --- | --- | --- | --- | --- |
|  | **core-front** | | **upstream-downstream** | | **core-front** | | **upstream-downstream** | |
|  | **p-value** | **pseudo-F** | **p-value** | **pseudo-F** | **p-value** | **pseudo-F** | **p-value** | **pseudo-F** |
| hemolymph | 0.233 | 1.24798 | 0.038 | 1.97748 | 0.211 | 1.33254 | 0.003 | 4.43042 |
| intestine | 0.017 | 2.18175 | 0.359 | 1.03873 | 0.159 | 1.59869 | 0.334 | 1.05177 |
| sediment | 0.01 | 2.22431 | 0.003 | 2.80071 | 0.044 | 3.07729 | 0.006 | 5.10009 |
| hepatopancreas | 0.255 | 1.13066 | 0.909 | 0.714999 | / | / | / | / |
|  | **unweighted UniFrac** | | | | **weighted UniFrac** | | | |
| **sample group** | **location** | | **p-value** | **pseudo-F** | **location** | | **p-value** | **pseudo-F** |
| hepatopancreas | / | | | / | UC | UF | 0.0120 | 4.0241 |
| exoskeleton | UC | DF | 0.0012 | 4.0579 | UC | DF | 0.0012 | 14.3280 |
|  |  | UC | 0.0050 | 1.7170 |  | UC | 0.0012 | 3.42621 |
|  |  | UF | 0.0012 | 6.6137 |  | UF | 0.0012 | 10.2810 |
|  | DF | UC | 0.0012 | 2.8002 | DF | UC | 0.0012 | 8.1536 |
|  |  | UF | 0.0012 | 5.0356 |  | UF | 0.0012 | 10.0947 |
|  | UC | UF | 0.0012 | 4.9368 | UC | UF | 0.0020 | 6.1673 |

**TABLE S4.** List of ASVs showing significantly different abundances between four sample locations. The analysis (ANCOM) was performed at phylum, family and genus levels. Locations with highest ASV abundances were determined based on the median number of ASVs.

| **ANCOM at phylum level** | | | | location with the highest ASV abundance |
| --- | --- | --- | --- | --- |
|  | Taxon | W-statistic value | clr mean difference |  |
| exoskeleton | k__Bacteria;p__Cyanobacteria | 28 | 53.452 | DF |
|  | k__Bacteria;p__Firmicutes | 25 | 8.997 | DF |
|  | k__Bacteria;p__OD1 | 21 | 14.345 | DC |
| hemolymph | no significant differences in abundance | | | |
| hepatopancreas | no significant differences in abundance | | | |
| intestine | no significant differences in abundance | | | |
| **ANCOM at family level** | | | | location with the highest ASV abundance |
|  | Taxon | W-statistic value | clr mean difference |  |
| exoskeleton | k__Bacteria;p__Firmicutes;c__Bacilli;o__Bacillales;f__Staphylococcaceae | 260 | 26.483 | DF |
|  | k__Bacteria;p__Proteobacteria;c__Gammaproteobacteria;o__Pseudomonadales;f__Moraxellaceae | 260 | 38.449 | DF |
|  | k__Bacteria;p__Cyanobacteria;c__;o__;f__ | 259 | 36.787 | DF |
|  | k__Bacteria;p__Cyanobacteria;c__Oscillatoriophycideae;o__Oscillatoriales;f__Phormidiaceae | 259 | 32.882 | DF |
|  | k__Bacteria;p__Cyanobacteria;c__Synechococcophycideae;o__Pseudanabaenales;f__Pseudanabaenaceae | 251 | 33.222 | UF |
|  | k__Bacteria;p__Firmicutes;c__Bacilli;o__Lactobacillales;f__Aerococcaceae | 241 | 22.85 | DF |
|  | k__Bacteria;p__Cyanobacteria;c__Oscillatoriophycideae;o__Chroococcales;f__Gomphosphaeriaceae | 240 | 61.247 | UF |
| hepatopancreas | k__Bacteria;p__Proteobacteria;c__Betaproteobacteria;o__Burkholderiales;f__Oxalobacteraceae | 93 | 10.196 | UC |
| hemolymph | no significant differences in abundance | | | |
| intestine | no significant differences in abundance | | | |
| **ANCOM at genus level** | | | | location with the highest ASV abundance |
|  | Taxon | W-statistic value | clr mean difference |  |
| exoskeleton | k__Bacteria;p__Proteobacteria;c__Gammaproteobacteria;o__Pseudomonadales;f__Moraxellaceae;g__Acinetobacter | 404 | 37.812 | DF |
|  | k__Bacteria;p__Firmicutes;c__Bacilli;o__Bacillales;f__Staphylococcaceae;g__Macrococcus | 404 | 26.75 | DF |
|  | k__Bacteria;p__Cyanobacteria;c__;o__;f__;g__ | 404 | 37.07 | DF |
|  | k__Bacteria;p__Cyanobacteria;c__Oscillatoriophycideae;o__Oscillatoriales;f__Phormidiaceae;g__Phormidium | 404 | 32.824 | DF |
|  | k__Bacteria;p__Cyanobacteria;c__Synechococcophycideae;o__Pseudanabaenales;f__Pseudanabaenaceae;g__Arthronema | 403 | 94.609 | UF |
|  | k__Bacteria;p__Firmicutes;c__Bacilli;o__Lactobacillales;f__Aerococcaceae;g__Aerococcus | 372 | 22.868 | DF |
|  | k__Bacteria;p__Cyanobacteria;c__Synechococcophycideae;o__Synechococcales;f__Synechococcaceae;g__Paulinella | 366 | 46.812 | UF |
| hemolymph | k__Bacteria;p__Proteobacteria;c__Alphaproteobacteria;o__Caulobacterales;f__Caulobacteraceae;g__Caulobacter | 112 | 8.284 | DC |
| hepatopancreas | k__Bacteria;p__Proteobacteria;c__Gammaproteobacteria;o__Pseudomonadales;f__Moraxellaceae;g__Psychrobacter | 217 | 16.231 | UF |
|  | k__Bacteria;p__Proteobacteria;c__Gammaproteobacteria;o__Enterobacteriales;f__Enterobacteriaceae;g__Salmonella | 216 | 14.351 | DF |
|  | k__Bacteria;p__Proteobacteria;c__Betaproteobacteria;o__Burkholderiales;f__Oxalobacteraceae;g__ | 210 | 21.505 | UC |
| intestine | no significant differences in abundance | | | |

**TABLE S5.** Number of sequenced and analyzed samples per site and sample type.

| A | number of samples in analyses of differences between all sample groups pooled together by site = 184 | | | | | |
| --- | --- | --- | --- | --- | --- | --- |
|  | **exoskeletal biofilm** | **hemolymph** | **hepatopancreas** | **intestine** | **sediment** | **water** |
| TOTAL | 39 | 41 | 33 | 48 | 17 | 6 |
| B | **number of samples in analyses of differences across invasion range (DF, DC, UC, UF) for each sample type = 173** | | | | | |
|  | **exoskeletal biofilm** | **hemolymph** | **hepatopancreas** | **intestine** | **sediment** | **water** |
| upstream front | 8 | 11 | 9 | 10 | 6 | excluded from these analyses |
| upstream core | 8 | 11 | 10 | 12 | 3 |  |
| downstream core | 12 | 11 | 9 | 12 | 2 |  |
| downstream front | 11 | 7 | 5 | 10 | 6 |  |
| TOTAL | 39 | 40 | 33 | 44 | 17 | 0 |
